# On Certain Methods of Treating Acute and Chronic Inflammations of the Eye, Lately Adopted at the Royal Westminster Ophthalmic Hospital

**Published:** 1828-09

**Authors:** G. J. Guthrie

**Affiliations:** Professor of Anatomy and Surgery to the Royal College of Surgeons; Surgeon to the Westminster Hospital, and to the Royal Westminster Ophthalmic Hospital, &c. &c. &c.


### THE LONDON
### Medical and Physical Journal.

N° 355, VOL. LX.] SEPTEMBER, 1828. [N° 27, *New Series*.

For many fortunate discoveries in medicine, and for the detection of numerous errors, the world is indebted to the rapid circulation of Monthly Journals; and there never existed any work, to which the Faculty, in Europe and America, were under deeper obligations, than to the *Medical and Physical Journal of London*, now forming a long, but an invaluable, series.—RUSH.

### ORIGINAL PAPERS,

AND

### CASES OBTAINED FROM PUBLIC INSTITUTIONS AND OTHER AUTHENTIC SOURCES.

### INFLAMMATIONS OF THE EYE.

*On certain Methods of treating Acute and Chronic Inflammations of the Eye, lately adopted at the* ROYAL WESTMINSTER OPHTHALMIC HOSPITAL. By G. J. GUTHRIE, F.R.S. Professor of Anatomy and Surgery to the Royal College of Surgeons; Surgeon to the Westminster Hospital, and to the Royal Westminster Ophthalmic Hospital, &c. &c. &c.

*To the Editors of the London Medical and Physical Journal.*

GENTLEMEN,—In transmitting to you the accompanying cases, illustrative of certain methods of treating chronic inflammation of the eye, I do not intend to notice at present the various trials which have been made during the last eighteen months, in order to arrive at the mode of proceeding now adopted. It will be sufficient to say, that in no instance has any evil resulted from the remedies employed; whilst in most cases they have been eminently serviceable. The principle on which they have been used has been that of exciting an action greater, and of a different nature, to that already existing in the part, and therefore they must have been powerful in their effects. I have found them most manageable in the shape of ointments, and I give the preference to the two following—

1. ℞. Argenti Nitratis, gr. ij. ad gr. x.; Liq. Plumbi Subacet. gtt. xv.; Ung. Cetacei ʒj.

2. ℞. Hydr. Oxymur. gr. iij. ad iv.; Liq. Plumbi Subacet. gtt. xv.; Ung. Cetacei ʒj.

The Argentum Nitratum, and Oxymuriate of Mercury, must be reduced first to an impalpable powder, then mixed with the ointment on a slab, and the Liquor Plumbi added. It may be done in a glass mortar. A double decomposition takes place in either ointment, which naturally diminishes the strength of each; but this change takes place slowly, particularly in the oxymuriate ointment, so that weeks elapse before they become inert. A very sensible difference is felt by the patient between an ointment only two days made, and another of two or three weeks' standing, and the stimulating qualities may be calculated according to the state of the eye, as well as the strength of the composition. The argentum nitratum ointment is grey when first made, but soon changes its colour to a brownish black. If the argentum nitratum be mixed with the ung. cetacei (as I once used it,) without the liquor plumbi, it dissolves more rapidly; when used, the powdered nitrate falls into the fold of the conjunctiva, or rests on the lid, and is apt to cause a slough, which is prevented by adding the lead.

The manner of using either ointment is by introducing between the lids a portion, larger or smaller as the case may seem to require it, from the size of a large pin's head to that of a garden pea. The eyelids, being closed, are to be rubbed gently with the finger, so as to diffuse the dissolving ointment over the whole surface of the conjunctiva: a part of it usually, however, works out by the motion of the lids, and should be wiped off (if the nitrate of silver,) to prevent its staining the skin. Both ointments cause pain: in some persons it is considerable, in others less so, lasting from half an hour to an hour and a half; and, when the ointment is newly made, sometimes for four hours, and even until the next day. On the subsidence of the pain caused by the ointment, that which previously existed is found to be relieved, if not entirely removed; and, on the subsequent day, the patient usually acknowledges the benefit he has received with regard to all the symptoms. When the application has been severe, and the patient very irritable, a state resembling white chemosis occasionally takes place, and appears formidable to a person unacquainted with the effect of the remedy: it soon, however, subsides. The eye should be fomented with warm anodyne fomentations.

I rarely repeat the application until the third day; but the feelings of the patient are the best guide, the return of some of the old sensations indicating the necessity for its use, which should be, if possible, anticipated. In some cases of acute inflammation, two or three applications will arrest the progress of a serious disease, and effect a cure. In chronic cases, the ointment must be continued for a considerable time, and occasionally alternated with other remedies. Where they create a state of regularly increased irritation, as they sometimes will do, cupping, purgatives, &c. are of service; when the remedies may again be resorted to.*

In the various trials I have made with these applications, and others of a similar nature, I have generally used purgatives, sometimes mildly, sometimes severely; and very often serious complaints have been treated successfully without any internal medicine. In some cases they disagree altogether, but then it is when they have been called upon to do that which ought not to have been expected from them. I do not consider them as specifics for all diseases, but as remedies capable of doing an infinity of good under proper management.

I have made these few observations in order to draw attention to them, and to the principle on which they are used. Any explanation which may be desired, or which curiosity may incite, will be given any Tuesday or Thursday morning, at half-past twelve o'clock, at the Ophthalmic Hospital, the doors of which have always been open to every practitioner.

I am, gentlemen, your obedient servant,

G. J. GUTHRIE.

P.S.—In the following cases I have suppressed the names of the different gentlemen whose care the patients had been under. It must, however, be understood they were all acquainted with, and professing a knowledge of, this branch of surgery. To those unacquainted with it, it may appear strange that persons who have been shewn to be curable should remain so many years under the best care, both in and out of hospital, with so little amendment. Nothing is, however, more common; and that persons labouring under diseases of this nature should require from twelve to twenty months in hospital before an approach to a cure is accomplished, the minutes of the Ophthalmic Committee of the House of Commons will abundantly testify. This fact will be a sufficient apology for the different trials which have been made, and for those which may yet be made, in order to discover better and speedier modes of cure.

* It is curious to see the feelings manifested by different persons. Some, indeed nearly all subjected to the use of these ointments at the Ophthalmic Hospital, asking to have them applied; others fearing the pain, but satisfied of the benefit received, and choosing their own days, and which eye, when both are affected.

Case I.—Maria Courage, 127, Long lane, Bermondsey, aged fifteen, has had her eyes bad for five years, so as scarcely to be able to see her way, and was frequently confined to the house for months together; was under the care of a gentleman professing a knowledge of diseases of the eye for about three years, going to him three times a week; was recommended by Mr. Furnival, of Westminster, to Mr. Guthrie, on the 9th of January, 1828, and has attended sometimes twice and sometimes once a week since that period.

On her first application, the conjunctiva of the palpebræ was thickened and granulated; that of the eyeball loaded with tortuous red vessels; the cornea, very opaque, having red vessels running in it, and several small ulcers on various parts of it; the cicatrices of others which had healed were very obvious; great intolerance of light. The Unguentum Hydrargyri Nitrat. was applied the first day, and has been repeated whenever she attended, save once, when, from having caught a little cold, it was omitted. The only internal medicine she has taken during this period has been senna and salts about once a fortnight.

She found benefit from the first application, and at the end of the first month was greatly relieved. She considers herself to have been well the last two months, although she has continued to attend. At present the only appearances of derangement are several small spots, the cicatrices of ulcers on the cornea, which cannot be entirely removed.

The following letter from her father will best explain his feelings on the subject.

Sir,—I return you many thanks for your kindness, working such wonders on my daughter's eyes; as I consider they are nearly well, after being bad nearly five years, I believe from the measles. She came to you the 9th day of January, 1828.

Your obedient servant,

THOS. COURAGE.

127, *Long lane, Bermondsey;*
*July 25th,* 1828.

Case II. by the Patient.

*A Sketch of the Cause and Progress of the Disease in my Eyes.*

The last week in April, 1822, I was sitting at work, a window being open over my head, a cold hazy day, the wind at north-east. (I mention this circumstance to explain that I have, since that period, invariably experienced a relapse when the weather was similar.) Sitting as above described, I caught cold in my left eye, and had a sensation as if sand had been in it. In two days it became swollen and closed; the ball appeared a mass of blood. I was advised to apply to Mr. —— : I waited on him accordingly. The fifth time of attendance, he told me I had lost my eye: he scarified the under lid, gave me a pill, and sent me home. By going to this institution I heard of you. I waited on you the next day: you pronounced it was an iritis, and remarked it was not so far gone but might be restored; by good fortune I made timely application to you. You ordered me to lose sixteen ounces of blood from the temple by cupping; to take two small pills every two hours, which caused a heavy salivation in two days, a sore mouth upwards of three weeks, which did away that great mischief, and saved my eye from total destruction.

In about five weeks after, the inflammation was communicated to the right eye, and ever since it has been the most troublesome and painful, always most susceptible of catching cold. The inflammation abated, and relapsed from time to time; the lids became granulated. I was rubbed with the sulphate of copper three times a week successively for two years, using various kinds of drops, repeated cupping and blistering, pills, &c. I was rubbed with alum and sulphate of copper occasionally for another year, which eventually cleared the eyelids, the sight gradually diminishing all the time. At length I could not see my way, nor discern any object distinctly, until the last five or six weeks, the stimulating ointment had the happy effect of clearing the apparently muddy fog that so long embarrassed my sight. I can now see every surrounding object quite distinctly.

P. J. WALSH.

*April 30th*, 1828.

I have all along observed the efficacy, or otherways, of the former applications.

P. J. Walsh was taken into the Westminster Hospital in December 1827, and was discharged cured 30th April, 1828, during which time the Ung. Argenti Nitratis was the only remedy made use of.

Case III.—Thomas Walsh, admitted a patient at the Royal Westminster Ophthalmic Hospital, March 23d, 1828; says he has had bad eyes more than five years. Has been under the care of Mr. —— three months, and subsequently under Mr. —— nine months; when Mr. —— said he need not attend him any longer, as he could do no more for him; that he might perhaps derive some benefit from an issue under each eye, but that he would not promise any great amendment. Walsh would not submit to the issues without the prospect of a cure, and left the institution in consequence. He then consulted several practitioners, was under some two, others three months, but found no relief. After this he applied to several quacks and advertisers, with as little effect. He then allowed two months to pass over without doing any thing, when he heard of Mr. Guthrie, and applied here in consequence.

On his admittance, there was much chronic inflammation of the cornea and sclerotica, both irides irregularly contracted, the right cornea very opaque, and considerable tarsal inflammation.

Same word for [illegible] and

*Treatment.*

March 22d.—℞. Hydrarg. Submur. gr. iij. h. s.; Magnes. Sulph. ℥j. mane.—Applic$^{r}$ Ung. Argent. Nitrat. to both eyes.

25th.—Rep$^{r}$ Pil. et M. S.—Rep$^{r}$ quoque Unguent. Arg. Nitr.

April 1.—Repetantur omnia. 3d. Rep$^{r}$. 8th. Rep$^{r}$. 10th. Rep$^{r}$.

After the first application of the ointment, he was much relieved; continued improving till the 10th of April, when the inflammation was all but removed, and the opacity of the cornea fast disappearing. He has been using the ointment up to this period, at first regularly, and afterwards once a week or occasionally. There is now no appearance of inflammation or opacity, and the irides are nearly natural.

*July* 31, 1828.

Case IV.—John Wade, aged forty-five, suffered an attack of inflammation of the right eye in February, 1827, which shortly after extended to the left: for which he was bled, blistered, and physicked by several gentlemen until October, 1827, when he applied for assistance at the Royal Westminster Infirmary for Diseases of the Eye. He was then unable to see his way, and was obliged to be led; the conjunctiva lining the lids was very much thickened and granulated, the cornea opaque, the conjunctiva of the ball very vascular, discharge, both watery and puriform, considerable. He was directed to use the Argentum Nitratum ointment, which in a short time relieved the most urgent symptoms; but, having to attend from Chelsea, was exposed to the frequent changes of the weather during the winter and spring, and suffered several attacks of acute inflammation.

On the 21st of June, 1828, he was admitted into the Westminster Hospital; was directed to be well purged, and to have the Ung. Argent. Nitrat. applied every third or fourth day, as his own feelings dictated. Under this treatment he gradually improved.

On the 2d July, five grains of the Pil. Hydrarg. were ordered to be taken every night, and some house aperient medicine in the morning.

August 2d.—The mouth is slightly sore from the pills, which are to be discontinued. The eyes have regularly improved since his admission into the hospital, and without any deviation; the corneæ are cleaner; the thickening of the lids is nearly gone, although the conjunctivæ lining them are still villous.

This case has been selected because it remains under treatment.

Case V. by the Patient.

Pearson Smith applied in January, 1828, to Mr. Guthrie, having been six years suffering from sore eyes, for which he had sought relief in vain from many gentlemen; and was then so nearly blind as not to be able to see a post. The black ointment was used, with almost instant relief, (the Ung. Argent. Nitrat.); attended regularly the first two months, afterwards at intervals, until April, when, thinking himself well, he went to work. Suffering a slight relapse in June, has again attended, and feels himself nearly well. Considers he owes his cure to the black ointment alone.

Case VI.—Ann Adnam, aged thirteen, has been unable to open or use her eyes until lately for the last twenty-two months, although she had been constantly under treatment at the Royal Westminster Ophthalmic Hospital for the first year. She was then put under the care of other persons; but, finding her eyes getting worse, she was readmitted into the Ophthalmic Hospital, April 8th, 1828, and had the Argent. Nitrat. ointment applied, which has been continued twice a week ever since, with an occasional dose of calomel, with salts and senna. She can now open the eyes; the corneæ are much clearer, and she can see. She is very subject to relapses on the slightest cold, but there is now every appearance of her getting well. Until the Ung. Argent. Nitrat. was applied, no other remedy seemed to be of the least use.

*July* 31, 1828.

Case VII.—Thomas Porter, aged eighteen, has been suffering from chronic inflammation of the eyes, more or less, for the last five years, and particularly for the last two, so as to be unable to work or get his bread; applied at the Ophthalmic Hospital, 24th July, 1828, in this state. The Unguent. Argent. Nitrat. was used on the 24th, 26th, 29th, and August 2d; on which day he says, " he considers himself nearly well; the pain is entirely gone, and he can see a great deal clearer." The morbid vascularity of the eyes has disappeared, but the corneæ bear the cicatrices of several ulcers.

Case VIII. *Acute Inflammation.*—William Bacher, aged thirty-four, applied February 26th, 1828, with acute catarrhal inflammation, of three days' standing in the right eye, and two in the left. It began with itching, followed by pain, as if something was in the eye, attended by a discharge of hot water, which prevents his sleeping, from the quantity which fills the eye, and forces him to open the lids with his fingers; cannot bear the light, and there is a difficulty in opening the eyelids, from the thick matter which in the night glues them together; pain in the head and over the eyes; the right suffused of a yellowish colour, and streaked with red vessels, arborescent, patchy with slight extravasation; some vessels running straight up to the cornea, others arborescing; streaks of mucus in the folds of the conjunctiva; edges of the lids slimy. Separating the lids relieves the uneasy sensations.

**The Ung. Argenti Nitratis gr. xv. ad ʒj. applied.—No internal treatment.**

27th.—The pain from the ointment lasted until seven in the evening, (six hours); discharged a good deal of water from the eyes in the night, but was much easier, as there was very little matter after seven in the evening; they therefore stuck together but slightly, nothing in comparison with the night before. There is now the same intolerance of light; but little discharge of water; very little sandy feel, or pain, perhaps once an hour; eyes are not so red, but the redness is more in patches; headache better.

Apply warm water only.

28th.—Complaints all returned last night at twelve o'clock, and thinks himself as bad as ever: begs to have the ointment applied, which was done.

29th.—Is again better. Slept well last night; the eyes discharged water freely, but the lids did not adhere together; has little or no pain; bears the light better; conjunctivæ appear redder.

Apply the ointment gr. x. ad ʒj.

March 1st.—The ointment gave pain for three hours, but says he is much better, and slept well. No application.

2d.—Pain came on yesterday afternoon, although it did not prevent his sleeping well.

Apply the unguent.

3d.—Slept well last night. Free from pain, and has very little discharge; bears the light better; conjunctivæ red, but less so than hitherto, and more of a yellowish red. No application.

4th.—Right eye rather painful last night; left free from pain; both are better.

Repeat Unguent. Nitrat. Argent.

5th.—Nearly well, and wishes to go to his work.

10th.—Not quite well, but is obliged to work, having a large family.

Repeat the Unguent. Nitrat. Argent.

13th.—The Ung. Hydrargyri Nitratis to the eyelids at night.

18th.—Unguent. Argenti Nitratis.

April 1st.—Has not attended since the 18th. There is some slight chronic inflammation of the lids remaining, but thinks nothing of it.

N.B. Has had eight in the family affected in a similar manner, and all cured by the same means.—Lives 23, Cuctrop street, Westminster, and is in the employ of Mr. Morris, of Northumberland street.

Case IX.—William Meyers, aged fifteen. April 3, 1828; four days ill; others of the family in the same state. Outside of the upper lid red and swelled; conjunctivæ of a patchy redness; complains of an itchy, sandy feel, giving great pain when the eye fills with water, which it frequently does; the discharge feels gummy, and causes the eyelids to adhere; the conjunctivæ are red, and in patches; redness principally from the circumference; streaks of mucus in the folds of the conjunctivæ; cannot bear the light or fire.

**Unguent. Argent. Nitrat. to both eyes.**

5th.—Is better.—Repeat Unguent.

7th.—Continues improving.—Unguent. to the right eye only.

9th.—Repeat.

13th.—Relapse of inflammation in the right eye, which was getting well on the 9th; supposed from being absent.

**Unguent. Argent. Nitrat.**

15th.—Better.

19th.—Discharged cured, with others of his family, younger than himself, who were in a similar state.

Case kept by Mr. Pilkington. Lives 4, West street, Soho.

Case X.—Jane Simpson, aged twenty-eight, applied 10th April, 1828, having been ill one month, during which time she used warm water; complains of great pain in the eyes, with a discharge of hot tears and of gum (mucus), which has excoriated the skin of the surrounding parts; cannot bear the light.

**Apply Unguent Argent. Nitrat.**

12th.—Says she is well, and appears to be nearly so, having been cured by one application. She attended until the 13th May with the child.

Case XI.—Emily Simpson, aged nine months, has had sore eyes ever since she was born, for which the mother applied breast milk and rose-water ineffectually; admitted April 15th.

**Apply Unguent. Argent. Nitrat.**

13th.—Repeat. 17th.—Repeat. 24th.—Repeat.

29th.—Both eyes are now nearly well.

**Repeat Unguent.**

May 6th.—**Repetatur Unguent.**

13th.—Discharged well.

Case kept by Mr. Angus. Lives 41, Wellington street, near Manor street, Chelsea.

Case XII.—Ellen Forshaw, aged twenty-two, April 10, 1822; slight inflammation of the inside of the eyelids, with a hot watery discharge, and some mucous secretion, causing them to adhere at night; intolerance of strong light.

**Pil. Hydr. Submur. gr. vj.; Magnes. Sulph. ℥j.—Ung. Argent. Nitrat.**

17th.—Returned thanks, being well. Says the application of the ointment was painful, but it cured her.

Case kept by Mr. Pilkington. Lives in Mill street, Berkeley square.

Case XIII.—Barney Rowlands, aged thirty. April 17, 1828: inflammation of the conjunctiva of the right eye, of three days'

duration; there is considerable pain, and some intolerance of light. The disease seems disposed to extend to the cornea. Had inflammation of the left eye a short time ago.

Ungent. Argent. Nitrat. Magnes. Sulph. ℥j.

19th.—Nearly well.

Pil. Hydr. Subm. gr. vj. Magnes. Sulph. ℥j. Ung. Argent. Nitrat.

21st.—Well.—Case kept by Mr. Hall.

Case XIV.—Charles Fairhead, aged nineteen. May 10, 1828. Common inflammation of the right eye, and with ulceration of the cornea of the left.

Cucurb. c. ferro ad ℥xij. Pil. Hydr. Subm. gr. vj. Magnes. Sulph.— Hot water for fomentation.

12th.—Better.

Repeat Pil. et Magn. Sulph.

13th.—Right eye well; left worse.

Cucurb. c. ferro ad ℥xvj.—Rep^r Pil. et Magn.

15th.—There being little or no improvement, the Ung. Argent. Nitrat. was applied to the left eye; pills and salts repeated.

17th.—Repeat every thing.

22d.—The ointment has been of great benefit to the left eye.

Repeat.

27th.—Repeat. 29th, June 3d, 7th, 12th, discharged cured. Case kept by Mr. James.

[To be continued.]

